# Gynecological and Obstetrical Society of Baltimore

**Published:** 1891-06

**Authors:** William S. Gardner

**Affiliations:** Secretary; 712 N. Howard street


### GYNECOLOGICAL AND OBSTETRICAL SOCIETY OF BALTIMORE.

### MARCH MEETING.

The President, Dr. Henry M. Wilson, in the chair.

Dr. Howard A. Kelley read a paper upon the Technique of the Cæsarean Section.

The Porro operatiou was rejected, excepting under special peculiar circumstances; for example, when there was good reason to suspect septic tnfection, as after prolonged efforts at delivery, at turning, or the use of the forceps, also in cases of large tumors occupying the body of the uterus, or in some cases of cancer or in uncontrollable hemorrhage from the placental rite. Thus limited, the conservative operation and the Parro operation are mutually exclusive, not occupying the same field.

It is a serious surgical error to mutilate a woman by performing the Parro operation where special indications do not exist.

In a healthy case, free from sepsis, with unruptured membranes, it is not necessary to deliver the uterus from the abdomen before incising it and delivering the child. It is rarely necessary to use any constricting ligature around the cervical end of the uterus. Excessive hemorrhage from the placental site or the margin of the wound can very well be temporarily controlled by constricting the cervix with the hands of an assistant.

The uterine suture consists of deep sutures, embracing the peritoneum and muscularia, but not the decidua. About ten such sutures are needed. Between each of these deep sutures, half deep sutures can be passed, securing perfect coaptation of the peritoneal surfaces. The sero-serus sutures are not necessary in cases free from any suspicion of infection. In such clean cases, the uterus is dropped back into the abdomen. If there exists a slight suspicion, it is of advantage to draw the omentum down behind the uterus, thus favoring the discharge of any septic material through the lower angle of the wound.

Drainage of the pelvic cavity cannot be efficiently carried out. The abdominal wound must be concealed by a dressing made of cotton dissolved in alcohol and ether, containing one part bichloride to 16,000. A little strip of gauze is laid over the wound, saturated with this solution. This adheres until it is time to take the sutures out, concealing the wound, and preventing contamination from the outside much better than many layers of gauze and cotton. The baby should be allowed to nurse as soon as the mother has thoroughly recovered from the anaesthetic.

The vagina should not be douched out as a matter of routine. The vaginal outlet should be secured from the introduction of sepsis from without by separating the labia and throwing into the vulvar orifice a drachm of powdered iodoform and boric acid (1 to 7). A cotton pad loosely applied to the vulva should be changed as often as soiled by the discharges.

Dr. Chas P. Noble : In the technique of the operation laid down by Dr. Kelly, reference has been made to typical cases. In such cases I agree entirely with what he has said. But all cases are not typical. I will report an unique case upon which I did the Caesarean Section recently.

Dr. Kelly had operated in a previous pregnancy. As a result of the first operation there remained a fistula opening from the uterine cavity through the abdominal wall. Notwithstanding this fistula, she became pregnant, and for several weeks the amniotic bag protruded into the opening, so that there was nothing between the foetus and the outer world but the thin amniotic sac.

This sac ruptured at the thirty-third week. The woman had a generally contracted pelvis; besides having a large mass of ec tissue behind the cervix, left from her previous Caesarean labor. Had spontaneous labor been possible, the foetus would have escaped through the fistula and not per vaginam. In view of the conditious, I thought Caesrean Section preferable to delivering the mutilated foetus per vias naturales.

The finger was inserted into the uterus through the fistula, and with this as a guide, the incision was made through the utero-abdominal walls.

Sufficient room not being afforded for delivery the peritoneal cavity was opened and the uterus incision lengthened. The living foetus was then delivered.

The placenta and membranes were firmly adherent, and were slowly pealed off. To control bleeding during this time it was necessary to insert the uterus through the abdominal incision to enable the assistant to grasp the lower segment.

The patient passed through a normal puerperium and is quite well.

Three cases of Caesarean section have been observed by me, all having made good recoveries. When the operation is done at the proper time, and after the method described by Dr. Kelly, I am sure this result will be quite uniform.

The essentials of success are:

1. Operation at the proper time, before labor, and the beginning of labor.
2. Rapidity in operating.
3. Accurate suturing.
4. Asepsis.

With reference to suturing, I believe that the Lembert suture as ordinarily described, is purely theoretical. The peritoneum will not hold a suture. Operators have unconsciously included the deeper tissues in the so-called Lembert suture. An important point, not generally recognized is, that the diagnosis should be made in the last weeks of pregnancy, and the operation be done at the close of pregnancy before labor sets in, or immediately thereafter.

Dr. B. B. Browne: I think all the procedures recommended are in the main correct, and are in accordance with the rules and suggestions laid down five or six years ago by Garrigues, Savenger and Leopold; these should be carried out in ideal cases, but unfortunately we meet with many complications which must be dealt with as they occur.

Having recently performed the operation myself and looked up the literature and technique of the subject, I was surprised to find that we can to-day make but little improvement or change for the better.

Dr. T. A. Ashby: I wish to congratulate Dr. Kelly on his brilliant success with the Caesarean section. This success is convincing proof of what can be done when the section is instituted under proper conditions and at a proper time.

Dr. W. P. Chunn: I did not hear the first part of the history of the case, but think I would have removed the ovaries or tied the Falloppian tubes to prevent future conception.

Dr. Noble: In doing a Caesarean section, I would not touch the ovaries and tubes as Dr. Chunn speaks of doing, but would do nothing to prolong the operation. Tying of the tubes would probably cause Salpiongitis. This objection is purely theoretical. So far as I know, this has been done only twice; once in England and once in America.

Dr. Brinton: I have been for some years interested in measuring the pelvis of women. Veiy often we go to labor cases without knowing anything about the condition of the pelvis.

With the hospital surgeon who has the best facilities, the Caesarean operation will undoubtedly be the best in cases of extreme pelvic contraction. But with the average practitioner, what is best? I think that with these physicians that craniotomy will hold the place.

In speaking of craniotomy "holding its place," I referred to those cases of pelvic contraction where the child could be extracted without harm to the mother, say from 1 3-4 to 3 inches.

Dr. T. A. Ashby: I must offer an apology for presenting a series of experiences which are familiar to all who have done much intra-abdominal work.

I have brought these charred remnants of tubal and ovarian inflammation before the Society to invite discussion, not to exhibit anything original. They represent nearly every phase of intra-pelvic inflammation, and illustrate the various degenerative conditions which are found in the pelvis after an inflammatory fire has passed over their tissues. Of the nine experiences here presented, removed from the same number of cases, no two are alike.

In one case the tube has received the brunt of the attack, in another the ovary is involved, whilst in a third both tube and ovary are tied up in a knot by adhesive inflammation, and so on through the series.

The clinical histories of these cases would be exceedingly interesting, did time admit of a recital, but I shall not tax your patience with details.

We have the same old story in all of these cases, save two—one the large specimen of a tubal sac of uncertain origin, probably an interrupted tubal pregnancy of long standing, and the other the remnants of a catarrhal salpingitis and ovaritis with intra-pelvic adhesions. Of the other seven specimens the origin of the condition is of chief interest in this connection since they explain to my mind the essential factor in the production of the specimen here presented. Each of these women have borne one or more children; in each case the history of the intra pelvic trouble dates from the last lying-in period, which was accompanied with mild or severe symptoms of child-bed fever. In each of these women there was an old lacerated cervix ; in some, more pronounced than in others. The histories of these cases, as far as they can be made out, and can be interpreted, tell the simple story. During labor, a cervical tear occurred, in this wound septic material gained a lodgement, a septic process was established which extended from the cervix to the cavity, from the cavity to the tubes and from the tubes to the intra-pelvic peritoneum.

The severity of the symptoms in each case must have borne some relation to the septic process and the tissues involved.

These cases illustrate the fearful havoc which a septic process following parturition may occasion among the pelvic organs. A little fire kindleth a mighty conflagration, is literally true in more respects than one. In an experience with other cases, I have observed this septic process in its very beginning when limited to the cervix and cavity, and I have seen the lying-in woman's temperature fall from 103 degrees to normal, within twelve hours after thorough cleaning and disinfection of the cervix and cavity in these cases and a complete arrest of the process before the tubes were involved. In another case I have seen tubal and general pelvic-peritonitis in active force following immediately the infection in the cervix and cavity. This experience convinces me, despite all other theoretical teachings, that we have in the lying-in state, an explanation of those intra-pelvic diseases which render the lives of so many women useless and oftentimes utterly miserable. Now, is it necessary that the lying-in period should be surrounded with extra hazard, high temperature, and severe pain?

The lesson clearly taught by such experience is, that aseptic conditions should be enforced in every case of labor, that the least suspicion of sepsis should lead to immediate investigation of the uterine cervix and cavity with a view to thorough cleaning and arrest of the septic process. If this be done, as I have done it in a number of cases seen with medical friends in consultation, we can cut short asepsis and arrest a condition which will surely extend to the tubes and pelvic peritoneum in the absence of prompt attention.

Dr. J. Whitridge Williams: The specimen exhibited represents a class of cases that are very common, and which will become more so as we become more expert in bimanual examination. Indeed, to a skillful palpator it almost seems that the majority of women examined have more or less tubal or ovarian disease.

The etiology in many cases is doubtful, but most observers appear to cling to Nœgerrath's theory of latent gonorrhœa. Examination of the pus in cases of pyosalpingx brings forward most interesting facts. For in most cases it is impossible to discover any species of bacteria, either under the microscope or by culture methods, which shows that the bacteria which caused the trouble have long since died, for closed pus cavities are not particularly favorable for the growth of organisms.

Clinically, the cases due to the pus organisms are much more acute and virulent than those due to the gonococcus. These results correspond with those of Zwerfel, of Leipzig, who has just published his observations. He also found the gono and streptococcus, but not the staphylococcus. In one of his streptococcus cases, the subject was an undoubted virgin, and he accounted for the infection by an abscess following an attack of typhoid fever some years before.

A close study of the clinical history of a number of cases inclines me to believe that the majority of cases follow infection during labor or after an incomplete abortion; for in many cases it is impossible to obtain even a history of leucorrhœa before labor, which would apparently exclude gonorrhœal infection.

By infection during child-birth, I do not necessarily mean the cases in which we have well marked puerperal fever, but the milder degrees of infection as well; for most of the cases of so-called milk fever are due to infection, and may give rise to serious results.

Zweifel, on the contrary, who bas just published a remarkable series 79 salpingo-oophorectomies, with only one death, believes in the gonorrhœal origin of most cases. Saenger traces most of the cases in virgins back to a gonorrhoeal salpingitis during childhood, which has persisted and ultimately affected the fallopian tube. While I do not feel justified in subscribing to this view, I can say that it is quite probable. For lately I have seen quite a number of cases of undoubted gonorrhoea in little girls of from two to seven years of age, in which there was no suspicion of criminal action.

In eight cases of vaginitis in little girls which I have examined, I found gonococci in six of them. In several the mode of infection was quite clear. In one case the husband acknowledged an attack of gonorrhoea, with which he infected his wife during her pregnancy, and each of the children born after it had opthalmic neonatorum, followed, when they were older, by gonorrhoeal vaginitis. In another case, an older brother had gonorrhoea, and his two little sisters had used his towels in bathing.

Dr. Brinton: I can corroborate the views of Dr. Williams in regard to the specific origin of the cases of vaginitis in children. Having recently treated, first, the father, with gonorrhoea; later, the mother, and in a fortnight from the time the father consulted me, was called to see the little daughter, aged four, with a severe "vaginitis," which yielded to the usual treatment in about the usual time. My experience has been, that if a child is found with a "vaginits," close investigation will prove that some older member of the family has either a "urethral" or a "vaginal" discharge.

Dr. Noble: Dr. Ashby has brought up so many points that it is difficult to know just what to take up.

It is now the fashion to call all unilatereal collections of blood extra-uterine pregnancies. But I have recently had a case that proved not to be a pregnancy. With reference to the uterine hemorrhage coming from the tubes, we do know as a fact that it is possible for blood to come from the tubes. This was common to all in the days when the stump was treated by the extra-peritoneal method in doing ovariotomy. I am quite sure that gonorrhoea has been the cause of most of the cases of pyosalpinx that I have seen, and I think the cause of salpingitis in young women is often from simple infection. Many cases of dysmenorrhoea in young women are due to salpingitis. In such cases it is unnecessary to question their chastity. I agree with all the speakers in reference to the relation of lacerated cervix to salpingitis.

Dr. H. P. C. Wilson: I did an explanatory laparotomy for a fibro-cystic tumor. In manipulation I found great tendency to bleeding, and as I could not get at the ovaries nor remove the tumor without causing death, I closed the abdomen. She got on well for fourteen hours, when she became very feeble, heart and respiration very weak. She was put upon digitalis and muriate of quinine and urea, but it did no good. The heart became so weak that the pulse could not be felt. I then began with five minims of tincture of strophanthus every three hours, and ether, m. xx, hypodermically, every three hours. The pulsebecame stronger, 125 to the minute, and she felt better. The next day she became unconscious, pupils dilated, face flushed, pulse 120, temperature normal. The medicine was with drawn, but she remained in this condition about twenty-four hours. To-day she is better, consciousness returning, pupils contracting. I have had no experience with the poisonous effects of strophanthus.

WILLIAM S. GARDNER, M. D., Secretary.

*712 N. Howard street.*

